# A Decade of Innovation: Lessons From Nationwide Dissemination of a Clinical Exercise Program for Older Adults

**DOI:** 10.1093/geroni/igaf122.485

**Published:** 2025-12-31

**Authors:** Katherine Hall, Megan Pearson, Rebekah Harris, Stephen Jennings, Tyara Mason, Kenneth Manning

**Affiliations:** Veterans Health Administration, Durham, North Carolina, United States; Durham VA Healthcare System, Durham, North Carolina, United States; VA Boston Healthcare System, Boston, Massachusetts, United States; Durham VA Healthcare Adminstration, Durham, North Carolina, United States; Durham VA Health Care System, Durham, North Carolina, United States; Durham VA Healthcare System, Durham, North Carolina, United States

## Abstract

The Gerofit program, established in 1986 addresses functional decline in older Veterans through tailored exercise. Initially piloted at 4 sites in 2013, it now operates at 33 VA medical centers. This abstract traces its dissemination via VA’s Geriatrics and Extended Care (GEC) Mentoring Partnerships Program and Office of Rural Health (ORH) Enterprise Wide Initiative. Expansion strategies included phased funding (GEC/ORH partnerships), virtual adaptations, standardized implementation training curriculum and toolkit development, and clinical note templates. The Gerofit National Coordinating Center (NCC) provided mentorship, monthly Community of Practice forum, and data infrastructure. Sustainability metrics tracked post-funding retention and program growth. From 2013–2025, Gerofit expanded 8-fold, serving >5,000 older adults annually. Telehealth programming (58% of current enrollees) and on-demand videos (119K views) increased rural access. Post-implementation, 94% of sites sustained programming through institutional buy-in. Key facilitators included leadership alignment with VA’s Whole Health initiative, multi-disciplinary provider teams, automated workload capture, and consumer demand for the program. Barriers included staff turnover, loss of clinical space, and competing facility priorities. Gerofit’s success reflects VA’s commitment to scaling evidence-based geriatric care. Hybrid delivery models and NCC support enable long-term viability and continued innovation, offering a blueprint for national dissemination of clinical wellness programs for medically complex older adults.

